# Precision Medicine in Inflammatory Bowel Disease: A Spotlight on Emerging Molecular Biomarkers

**DOI:** 10.3390/biomedicines12071520

**Published:** 2024-07-08

**Authors:** Antonio Mestrovic, Nikola Perkovic, Dorotea Bozic, Marko Kumric, Marino Vilovic, Josko Bozic

**Affiliations:** 1 Department of Gastroenterology, University Hospital of Split, Spinciceva 2, 21000 Split, Croatia; antonio.mestrovic1@gmail.com (A.M.); n.perkovic@yahoo.com (N.P.); dora.bozic@hotmail.com (D.B.); 2 Department of Pathophysiology, University of Split School of Medicine, Soltanska 2A, 21000 Split, Croatia; marko.kumric@mefst.hr; 3 Laboratory for Cardiometabolic Research, University of Split School of Medicine, Soltanska 2A, 21000 Split, Croatia

**Keywords:** biomarkers, inflammatory bowel disease, personalized medicine, fecal calprotectin, oncostatin M, metabolomics, microRNA, machine learning

## Abstract

Inflammatory bowel diseases (IBD) remain challenging in terms of understanding their causes and in terms of diagnosing, treating, and monitoring patients. Modern diagnosis combines biomarkers, imaging, and endoscopic methods. Common biomarkers like CRP and fecal calprotectin, while invaluable tools, have limitations and are not entirely specific to IBD. The limitations of existing markers and the invasiveness of endoscopic procedures highlight the need to discover and implement new markers. With an ideal biomarker, we could predict the risk of disease development, as well as the possibility of response to a particular therapy, which would be significant in elucidating the pathogenesis of the disease. Recent research in the fields of machine learning, proteomics, epigenetics, and gut microbiota provides further insight into the pathogenesis of the disease and is also revealing new biomarkers. New markers, such as BAFF, PGE-MUM, oncostatin M, microRNA panels, αvβ6 antibody, and S100A12 from stool, are increasingly being identified, with αvβ6 antibody and oncostatin M being potentially close to being presented into clinical practice. However, the specificity of certain markers still remains problematic. Furthermore, the use of expensive and less accessible technology for detecting new markers, such as microRNAs, represents a limitation for widespread use in clinical practice. Nevertheless, the need for non-invasive, comprehensive markers is becoming increasingly important regarding the complexity of treatment and overall management of IBD.

## 1. Introduction

Inflammatory bowel diseases (IBD) are chronic, idiopathic diseases that affect 4.9 million cases worldwide, with an estimated increase in the number of cases by 47.45% in a period from 1990 to 2019 [1]. The two main forms of IBD refer to Crohn’s disease (CD) and ulcerative colitis (UC), which primarily differ in clinical, pathophysiological, and histological features. Despite numerous investigations, the etiology of IBD still remains unclear. Based on current knowledge, inflammatory bowel diseases are the result of a combination of genetic predisposition, genome-environment interaction, changes in the microbiome, and consequent dysregulation of the immune system [2,3].

Given the wide range of differential diagnoses, the final diagnosis of IBD relies on a combination of clinical, endoscopic, and histological findings. The clinical course of inflammatory bowel disease is often marked by frequent relapses, necessitating detailed and long-term monitoring of patients’ conditions, including endoscopic imaging. This typically involves invasive methods, primarily colonoscopy. Treatment of patients with IBD has evolved over the past decades. Today, a personalized approach is increasingly advocated, requiring the use of complex and expensive drugs tailored to individual patients. Despite the introduction of numerous new drugs in treatment, the five-year cumulative risk of surgery remains at 7.0% for patients with UC and 18.0% for patients with CD [4].

The modern goal of treatment is not only symptom relief but to achieve both endoscopic and symptomatic remission, as presented through the updated version of the Selecting Therapeutic Targets in Inflammatory Bowel Disease (STRIDE) initiative, which outlines a treat-to-target strategy based on evidence and consensus by the International Organization for the Study of Inflammatory Bowel Diseases (IOIBD) [5]. Combining symptomatic and endoscopic remission is associated with better outcomes, a lower risk of relapse, a decreased need for corticosteroids, a reduced need for hospitalizations, lower rates of colectomy, and lower colorectal cancer risk [6,7,8]. Namely, the risk of developing colorectal carcinoma is at least twice as high in patients with inflammatory bowel diseases, which emphasizes an additional need for thorough monitoring and screening [9]. Furthermore, recent studies associate the achievement of endoscopic remission with greater success of biological therapy and a lower rate of relapse after withdrawal of anti-TNF therapy [10].

Additionally, studies showed outcome improvement when biomarkers are used in treatment decisions [11]. The CALM study, an open-label, randomized, controlled phase 3 study conducted in 22 countries, included patients with moderate to severe Crohn’s disease. Biomarkers used in the study were CRP and fecal calprotectin. It was the first study that concluded that timely escalation with an anti-tumor necrosis factor therapy on the basis of clinical symptoms combined with biomarkers in patients with early Crohn’s disease results in better clinical and endoscopic outcomes than symptom-driven decisions alone [11]. The frequent need to monitor the course of the disease, the treatment course, the assessment of mucosal healing, and the assessment of the risk of relapse dictate the need for non-invasive monitoring tools. A large cohort study in the USA showed that, within the first 24 months after initiation of biological therapy, monitoring (proactive or reactive) was performed in 56.4% of CD patients and 67.8% of UC patients, with considerable geographic variability [12]. However, early proactive monitoring of mucosal inflammation, primarily through endoscopy (performed in more than 87% of patients) within six months of biologic initiation, was associated with a reduction in disease-related complications over 24 months, mainly attributed to decreased steroid utilization [12]. However, results differ among studies. A retrospective population study, which included 39,734 newly treated IBD patients, revealed that fewer than half of patients underwent colonoscopy within 3 to 15 months after initiating new treatment. This indicates that the utilization of endoscopy for disease monitoring in clinical practice is insufficiently present [13].

The use of biomarkers so far has proven to be a practical solution, which avoids the need for invasive methods such as colonoscopy [8]. Namely, colonoscopy requires prior bowel cleaning, which often causes discomfort and reduces the patient’s compliance. Furthermore, colonoscopy requires an educated team, time, and adequate endoscopic equipment, especially regarding the availability of specific technologies such as chromoendoscopy or the narrow-band imaging (NBI) technique for the detection of dysplastic lesions, which are still not available in all centers. All of the above further impairs the quality of life of patients with IBD, emphasizing the need for more appropriate monitoring tools. In recent clinical practice, as well as scientific research, the use of biomarkers seems to bypass most of the mentioned obstacles and circumstances [8,14].

It is important to point out the necessity of patients’ inclusion in the decision-making process regarding the choice of the appropriate tool in the monitoring of the disease [15]. The results of a prospective study indicate that accuracy is the main criterion for IBD patients when choosing an adequate surveillance strategy, regardless of the invasiveness of the test. Namely, most patients were willing to choose stool-based testing over colonoscopy for disease monitoring only if the stool test was wrong at most 1 in 20 times [16]. Furthermore, there is a real need to identify biomarkers that are predictive of colorectal carcinoma (CRC) risk in patients with IBD [17]. Although the incidence of colorectal cancer in IBD patients has declined over the past 30 years, attributed to both successful CRC surveillance programs and improved control of mucosal inflammation, the risk of CRC remains significant. As surveillance programs heavily depend on colonoscopy, the extent to which colonoscopy can be replaced for CRC screening in IBD remains a subject of debate [18].

## 2. Biomarkers in General

Generally accepted definition of biomarker is: “a defined characteristic that is measured as an indicator of normal biological processes, pathogenic processes or responses to an exposure or intervention” [19,20]. Despite the simplified definition, the process of validating a new biomarker is very complex and includes analytical validation, qualification using an evidentiary assessment and utilization [20].

Biomarkers have become an indispensable tool in the management of patients with IBD [21]. Their usage can be seen in different levels: diagnosis, distinguishing between IBD and other diagnosis, predicting disease severity and activity, predicting treatment response, assessment of mucosal healing, monitoring drug-related adverse events and predicting recurrence of disease after therapy withdrawal [14,21]. Additionally, it appears that biomarkers may serve to identify those individuals who are at increased risk for developing IBD or those who already have subclinical pathology [14].

**Figure 1:**
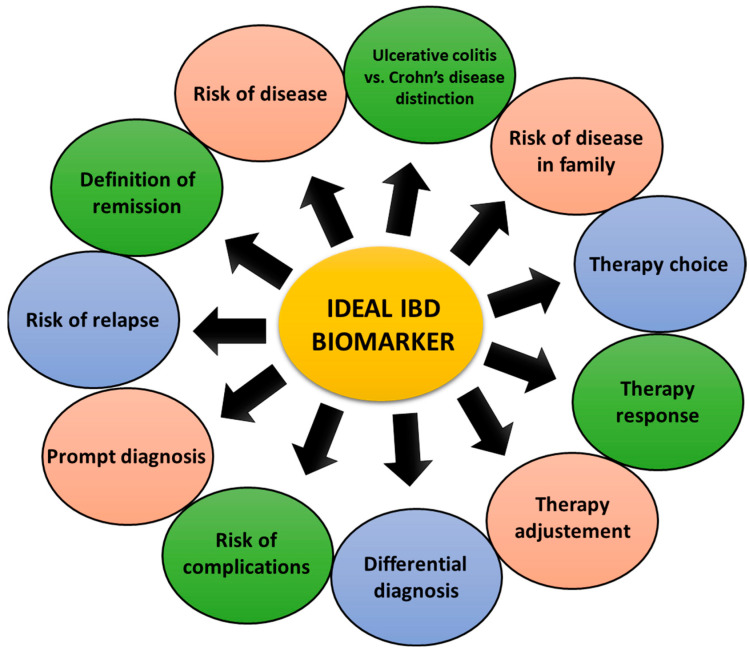
Expectations from ideal biomarkers in IBD.

Accurate selection of biomarkers represents a personalized approach in managing patients with IBD aiming optimal care and quality-life improvement. Ideal biomarker would be an accurate predictor at the moment of diagnosis of possible severity of the disease course to allow prompt intervention [22]. Figure 1 lists the expectations and requirements for an ideal biomarker.

As stated by Zilbauer et al., considering the purpose of use, biomarkers can be recognized as prognostic and predictive ones [23]. Prognostic markers are used to predict treatment-independent natural disease course [23]. By using these markers, we can avoid overtreatment in the mild course of the disease, thus reducing eventual adverse events. On the other hand, in severe disease courses, top-down treatment can be a favorable strategy, as suggested by current evidence [24,25,26]. PROFILE study was a multicenter, open-label, biomarker-stratified, randomized controlled trial that included adults with newly diagnosed active Crohn’s disease (Harvey-Bradshaw Index ≥ 7, either elevated C-reactive protein or fecal calprotectin or both, and endoscopic evidence of active inflammation) that received either top-down or accelerated step-up treatment [24]. Results of the study showed that top-down treatment (infliximab plus immunomodulator) achieved substantially better outcomes, including sustained steroid-free and surgery-free remission at one year, than accelerated step-up treatment [24]. Therefore, the authors of the study strongly emphasized top-down treatment as the standard of care for most patients as soon as possible after diagnosis [24].

On the other hand, predictive factors are used to predict short-term responses to a specific treatment, thus enabling modification of the therapeutic approach in case of lack of adequate response or harmful side effects [23]. Therefore, biomarkers can be used in all phases of the care of IBD patients (Figure 1) [27]. Current biomarkers used in IBD can be divided into serum, serological, and fecal biomarkers. Among them, CRP and fecal calprotectin stand out due to their frequent and practical use, but primarily as proven and reliable markers of disease activity [28].

In this review, we will present current and novel biomarkers in the context of their usefulness and limitations in evaluating disease activity, predicting mucosal healing, forecasting therapeutic response, and anticipating recurrence

## 3. Most Common Biomarkers in Clinical Practice

### 3.1. Serum Markers

#### 3.1.1. C-Reactive Protein (CRP)

CRP is an acute-phase reactant protein produced in hepatocytes in response to inflammatory cytokines, predominantly interleukin-6 (IL-6) [29]. CRP has a short half-life of about 19 h, which makes it the most responsive indicator of acute inflammation than most other acute phase reactants [29]. Elevated levels of CRP signify systemic inflammation and tissue damage, making it a valuable tool in diagnosing and assessing the activity of inflammatory diseases, including IBD. In the context of IBD, CRP levels indicate disease severity, response to therapy, and risk of complications. However, its fundamental role is differentiating between disease flare-ups and periods of remission. An elevated level of CRP is helpful in distinguishing mucosal active disease from quiescent IBD, while a CRP level < 10 mg/L indicates a remission stage of IBD [30,31]. Moreover, monitoring CRP alongside clinical symptoms is crucial in assessing the response to treatment, as well as in decision-making regarding treatment escalation, such as adjusting medication doses or transitioning to more potent therapies [30,31] (Table 1).

Persistently elevated CRP, despite treatment, may suggest treatment resistance or ongoing inflammation, prompting a reassessment of the therapeutic approach. Beyond its utility in diagnosis, therapy response, and monitoring, CRP serves as a prognostic marker for disease progression and complications in IBD [21]. Persistently elevated CRP levels during follow-up correlate with a higher likelihood of disease relapse and possible need for surgical intervention [22]. Meanwhile, high baseline CRP levels are associated with an increased risk of severe disease manifestations, especially in patients with Crohn's disease, such as strictures, fistulas, and bowel perforation [53].

Despite its clinical significance, CRP is not without limitations in the context of IBD management, mostly because CRP is not a disease-specific parameter [27]. Elevated levels occur in non-IBD enteritis, other inflammatory disorders, tissue damage or trauma, diabetes, intestinal, and other malignancies, thus limiting its diagnostic specificity in IBD [54,55,56,57]. Furthermore, it is important to address cases where CRP values remain normal despite active disease. This phenomenon, known as CRP discordance, is even more relevant in UC patients [27,32]. Studies have shown that approximately 50% of patients with active UC may have normal CRP levels during disease flares [30]. This discordance is less common in CD, where CRP tends to correlate better with disease activity [58]. The reasons for this discrepancy are not fully understood but may be related to genetic factors influencing CRP production or differences in the inflammatory processes between UC and CD [59]. Also, one of the possible explanations lies in the fact that inflammation in UC is confined in the mucosa, in opposition to transmural inflammation in CD [60]. Additionally, the extent and severity of intestinal inflammation can affect CRP levels, with more extensive disease generally associated with higher CRP values [58]. There is no satisfactory explanation for these differences, although serum IL-6 concentrations are reported to be higher in patients in patients with CD compared with UC and healthy controls [61]. Hence, it’s important for clinicians to be aware of this limitation when using CRP as a biomarker in IBD, especially in UC, and to consider other markers, such as fecal calprotectin or endoscopic evaluation, when assessing disease activity [32,62]. This underscores the need for a multi-faceted approach to disease monitoring in IBD rather than relying solely on CRP as an indicator of inflammation.

In conclusion, C-reactive protein plays a pivotal role in the evaluation and management of inflammatory bowel disease. Its association with disease activity, response to treatment, and risk stratification underscores its utility as a valuable biomarker in clinical practice. However, its nonspecificity and limitations mandate a multidimensional approach to IBD assessment, incorporating clinical evaluation, imaging studies, and endoscopic findings for optimal patient care. As our understanding of IBD pathogenesis and biomarker kinetics continues to evolve, CRP still remains a cornerstone in the collection of tools available to clinicians in the management of this complex disease entity.

#### 3.1.2. Erythrocyte Sedimentation Rate (ESR)

ESR, also known simply as sedimentation rate, represents a measurement of the rate at which red blood cells (erythrocytes) settle in a vertical column of blood over a specific period [27]. The mechanism underlying the increase in ESR during inflammation involves the elevation of plasma proteins, particularly fibrinogen, and globulins, which alter the viscosity and surface properties of blood, leading to faster sedimentation of erythrocytes [60]. In the context of IBD, elevated ESR levels often correlate with the severity of inflammation and disease activity. However, ESR can be elevated in response to any type of inflammation [27]. Unlike CRP, ESR is influenced by age, gender, pregnancy, anemia, polycythemia, various inflammatory conditions, and certain medications [27,33]. Moreover, CRP concentration changes faster in regard to disease activity [27] (Table 1).

ESR serves as a valuable biomarker in managing IBD, offering insights into disease activity, severity, and prognosis. Its simplicity, cost-effectiveness, and complementary role to other inflammatory markers make it especially useful. However, its interpretation should be contextualized within the broader clinical context, considering other relevant parameters and potential confounders.

#### 3.1.3. Leucine-Rich Alpha-2 Glycoprotein

Recent studies have shed light on the role of Leucine-Rich Alpha-2 Glycoprotein (LRG), an emerging biomarker and potential therapeutic target in IBD [21]. LRG, a 50-kDa glycoprotein, is predominantly synthesized by hepatocytes and secreted into the bloodstream in response to various inflammatory stimuli [21,63]. It belongs to the family of leucine-rich repeat (LRR) proteins, which are implicated in immune regulation and host defense mechanisms [64]. One of the key advantages of LRG as a biomarker is its production in response to numerous cytokines, such as TNF-α, IL-22, and IL-1β while being independent of IL-6. This characteristic enables LRG to provide a more comprehensive reflection of inflammation compared to CRP, which is primarily induced by IL-6 [21]. This broader inflammatory response could potentially make LRG a more versatile biomarker for IBD. However, it’s important to note that this wider range of inflammatory stimuli might also lead to reduced specificity in certain scenarios, a limitation that warrants further investigation. Studies show that LRG is more representative of intestinal inflammation than CRP, likely because it is derived from cytokine-stimulated neutrophils and intestinal epithelial cells [34]. This gut-specific origin of LRG could provide a more accurate reflection of intestinal inflammation compared to systemic markers like CRP. However, comparative studies directly assessing the sensitivity and specificity of LRG versus CRP and fecal calprotectin in detecting active disease are needed to fully establish its superiority.

Previous studies have shown that LRG levels are high in active inflammatory bowel disease, both in UC and CD, with a decrease in more stable disease [35,65]. Furthermore, LRG levels appear to correlate more accurately with clinical and endoscopic scores in active UC and CD compared to CRP. Notably, LRG can even predict mucosal healing in patients who have normal CRP levels [35,36,37,38]. LRG has been shown to be a valuable factor in evaluating small intestine mucosal healing, especially in combination with ileocolonoscopy [66] (Table 1). This ability to detect subclinical inflammation in patients with normal CRP levels could potentially change clinical practice by allowing for earlier intervention and more precise monitoring of disease activity. However, the implications of this capability on long-term outcomes and treatment strategies need to be further explored.

In a prospective study including 227 patients, Takenaka et al. concluded that LRG is a highly accurate serum biomarker for detecting transmural activity in patients with CD [39]. The results showed a positive correlation between LRG and the total magnetic resonance enterography (MRE) score in the context of the detection of transmural inflammation, with LRG being more accurate than CDAI and CRP [39]. While these findings are promising, it’s important to critically analyze how the accuracy of LRG compares to other methods of detecting transmural inflammation, such as ultrasound or histological assessment. Moreover, patients with high LRG levels were also strongly associated with CD-related hospitalization, surgery, and clinical relapse compared with those with low LRG levels, with *p* < 0.01 for all situations [39]. This prognostic value of LRG could have significant implications for clinical decision-making and risk stratification in CD patients. However, future studies should investigate how this prognostic information compares to other established risk factors and how it might be integrated into clinical practice algorithms.

In conclusion, while LRG shows great promise as a biomarker in IBD, further research is still needed to fully elucidate its role in different clinical scenarios, its comparative performance against established biomarkers, and its potential impact on treatment decisions and long-term outcomes.

### 3.2. Serological Antibodies

Perinuclear antineutrophil cytoplasmic antibodies (pANCA) and anti-Saccharomyces cerevisiae antibodies (ASCA) are the two main antibodies currently used and examined in IBD [27]. Serological antibodies associated with IBD predominantly target microbial and self-antigens, reflecting the dysregulated immune response characteristic of the disease [67]. Among the most studied serological markers in IBD are anti-Saccharomyces cerevisiae antibodies (ASCA), which target various components of the yeast cell wall [68]. ASCA has been consistently associated with CD, with approximately 60–70% of CD patients testing positive for these antibodies [40,42]. However, they can be found to be positive in 10–15% of patients with UC and in less than 5% of patients with non-IBD colitis [40,42] (Table 1). Although not as valuable as a diagnostic tool, ASCA has been shown to be a possible predictive factor in the disease's course. A meta-analysis, which included 24 studies, indicated that positive ASCA status is a risk factor for early-onset age, ileal involvement, complicated behavior, perianal disease, and requirement for surgery in CD [40]. This suggests that ASCA testing could potentially aid in risk stratification and treatment planning for CD patients.

Another significant serological marker in IBD is a perinuclear antineutrophil cytoplasmic antibody (pANCA), which is induced by a cross-reaction with intestinal bacterial antigens [67]. The antigen that corresponds to pANCA is thought to be histone 1 [67]. pANCA positivity is more commonly associated with UC, although it can also be detected in a subset of CD patients [42]. It has been shown that p-ANCA has a sensitivity of 52% and a specificity of 91% in distinguishing UC from CD [41] (Table 1). However, it’s important to note that pANCA can also be detected in a subset of CD patients, which may complicate differential diagnosis in some cases.

Interesting findings have been associated with proteinase 3 antineutrophil cytoplasmic antibody (PR3-ANCA), mostly known as a marker for granulomatosis with polyangiitis [68]. Imakiire et al. showed that PR3-ANCA measurement is useful not only for diagnosing UC but also for evaluating disease severity and extension and predicting the clinical course [69]. Results of the study showed that PR3-ANCA at level ≥ 3.5 U/mL demonstrated 44.5% sensitivity and 95.6% specificity for the diagnosis of UC, with PR3-ANCA positivity more prevalent in new-onset UC patients (58.4%), [69]. Furthermore, the disease severity and extension were more severe in PR3-ANCA positive patients than in the PR3-ANCA negative group (*p* < 0.001), with the proportion of patients who required steroids for induction therapy significantly higher among the PR3-ANCA positive than in the negative group [69]. These findings suggest that PR3-ANCA could serve as a valuable biomarker for disease activity and treatment response in UC.

While these serological markers show promise, it’s crucial to interpret them in the context of clinical presentation and other diagnostic tests. The moderate sensitivity of individual markers limits their standalone diagnostic value. However, combining multiple markers or using them in conjunction with other clinical and endoscopic findings may enhance their utility in IBD diagnosis and management. Future research should focus on identifying novel antibodies and developing multi-marker panels to improve diagnostic accuracy and prognostic capabilities in IBD.

### 3.3. Fecal Biomarkers

Fecal biomarkers commonly include calprotectin, lactoferrin and S100A12. All of them are metal chelating agents with antimicrobial activity that are overly expressed in inflammatory conditions due to active secretion or spontaneous release from necrotic immune cells [43].

#### 3.3.1. Calprotectin

Calprotectin is a glycoprotein (36-kD) found primarily in neutrophil’s cytoplasm, as well as in various other immune cells such as monocytes and macrophages, and is released at the site of inflammation [43]. It can be isolated from stool samples that should be collected from the first morning stool [70]. The molecule remains stable for 3–7 days at room temperature and even for a year when stored at −20 °C [43].

Calprotectin may be used as a biomarker in cases of suspected IBD, in differentiation from irritable bowel syndrome (IBS), in monitoring of disease activity, predicting remission, monitoring response to anti-TNFα therapy, and detecting postoperative recurrence in CD [44]. Most importantly, calprotectin level monitoring can be used to predict disease progression or to confirm the quiescent phase in an individual patient's management, thereby bypassing unnecessary invasive endoscopic procedures [43] (Table 1).

According to the review by Laserna-Mendieta et al., published in 2019, 13 meta-analyses have been conducted in order to determine calprotectin performance in IBD [44]. Eight of them assessed calprotectin values in the diagnosis of IBD, two of them evaluated the role of calprotectin in monitoring disease activity, one assessed its value in the prediction of relapse, and the final two evaluated its role in the prediction of postoperative recurrence in CD [44]. A meta-analysis that included the largest number of patients (1267), with both UC and CD, determined a cut-off value of 50 µg/g in the diagnosis of IBD in adults and children, with a sensitivity of 0.89 and a specificity of 0.81 [71]. Three years later, another study determined a cut-off value of 24–150 µg/g with a sensitivity of 0.93 and specificity of 0.96 [72].

A recent meta-analysis was published in order to evaluate the diagnostic performance of calprotectin in the assessment of endoscopic activity in adults. The authors determined a cut-off value of 50 µg/g (sensitivity 0.90) in the detection of endoscopically active disease on 2822 patients with IBD [73]. However, they found the best specificity (0.78) at cut-off levels >100 µg/g [71]. Similarly, Lin et al. evaluated the performance of calprotectin values in disease monitoring and determined a cut-off value of 250 µg/g (sensitivity 0.80, specificity 0.82) in the meta-analysis that included 1471 patients with IBD [74]. Moreover, another meta-analysis of six studies (672 adults with IBD) was conducted to predict relapse in patients with IBD and reported a wide cut-off range (50–340 µg/g), with a pooled sensitivity of 78% and specificity of 73% [75], while Shi et al. included 24 prospective studies, and also evaluated the diagnostic accuracy of calprotectin in predicting IBD relapse [76]. The authors found an optimal cut-off value of 152 µg/g (sensitivity 0.720, specificity 0.740) and concluded that calprotectin is a useful and inexpensive biomarker for accurate early prediction of IBD relapse [76]. Several experts found that calprotectin values correlate better with the disease activity in UC than in CD, primarily due to the fact that CDAI as a clinical score may not detect the subclinical, covert phase of relapse [46]. This explanation is supported by the fact that Sipponen et al. found a strong correlation (r = 0.729, *p* < 0.001) of calprotectin when using a purely endoscopic index (CDEIS, Crohn’s Disease Endoscopic Index of Severity) to assess CD activity [5,47].

Certain authors evaluated the value of calprotectin in differentiating IBD from IBS and found that normal calprotectin levels have a very high negative predictive value for IBD [45,68]. Therefore, this simple and non-invasive marker may help avoid unnecessary endoscopies in patients for whom the diagnosis of IBD is unlikely. Thus, it serves as an important and cost-effective biomarker. However, while levels above 250 ug/g would definitely require endoscopic evaluation, levels between 150 µg/g and 250 µg/g are considered a grey zone according to STRIDE-II guidelines and usually present challenges in clinical decisions [5]. Calprotectin values above 50 µg/g have a low positive predictive value for differentiating IBD from IBS. In contrast, a calprotectin level of ≤40 µg/g indicates a ≤1% probability of having IBD and generally excludes the diagnosis [77]. Finally, since the rise in calprotectin levels is always caused by an inflammatory process in the colon, it is always important to exclude acute gastroenteritis or enterocolitis with stool cultivation at the beginning of clinical evaluation.

#### 3.3.2. Lactoferrin

Lactoferrin is a glycoprotein (80 kDa) with antimicrobial properties present in the granules of neutrophilic granulocytes and secretory epithelia [43,68]. The molecule may remain stable for up to 7 days at room temperature or stored at 4 °C (60,73). This stability is a significant advantage for clinical use, allowing flexibility in sample collection and transport. Lactoferrin is a fecal biomarker that indicates intestinal inflammation and correlates well with the endoscopic and histologic disease activity in patients with IBD [46]. This correlation suggests its potential utility in non-invasive disease monitoring; however, the strength of this correlation may vary between UC and CD, warranting further investigation. A recent meta-analysis by Dai et al., examining 10 studies encompassing 773 patients, reported promising diagnostic accuracy for fecal lactoferrin in assessing IBD activity. The analysis revealed pooled sensitivity and specificity values of 81% and 82% for UC and 82% and 71% for CD, respectively. These findings suggest that lactoferrin may be particularly effective in evaluating UC activity, though its performance in CD is also noteworthy. The authors concluded that lactoferrin represents a simple, cost-effective marker for assessing IBD activity, with a potential edge in UC patients [48]. Sipponen et al. found a strong and significant correlation (r = 0.773, *p* < 0.001) between fecal lactoferrin levels and the CDEIS, as well as endoscopic findings in patients with CD. They proposed a cut-off value of 10 µg/g for determining disease activity, with a sensitivity of 66%, specificity of 92%, positive predictive value (PPV) of 94%, and negative predictive value (NPV) of 59% (Table 1) [47]. While these results are promising, the relatively low sensitivity and NPV suggest that lactoferrin alone may not be sufficient to rule out active disease in all cases.

Yamamoto et al. investigated the accuracy of lactoferrin in predicting IBD relapse and proposed a cut-off value of 170 ug/g (sensitivity 76%, specificity 76%), although authors did not find a statistically significant difference between relapsed and nonrelapsed patients [43,49]. This lack of statistically significant difference between relapsed and non-relapsed patients raises questions about its predictive power and highlights the need for larger, prospective studies to validate these findings. It’s important to note that not all studies have found lactoferrin to be superior to other biomarkers. Another meta-analysis assessed the utility of several biomarkers in order to exclude IBD in adults with IBS. Two of the studies included in the analysis assessed the efficacy of lactoferrin and did not find any significant clinical utility compared to CRP and calprotectin [77]. This underscores the importance of considering lactoferrin in conjunction with other clinical and laboratory parameters rather than as a standalone diagnostic tool.

In conclusion, while fecal lactoferrin shows moderate performance in IBD patients, particularly in UC, its clinical utility is not yet fully established. Further research is needed to define optimal cut-off values for detecting clinically active disease and predicting early relapse across different IBD phenotypes. Additionally, studies comparing lactoferrin head-to-head with other fecal biomarkers like calprotectin could help clarify its relative strengths and limitations in IBD management.

#### 3.3.3. S100A12

**Figure 2:**
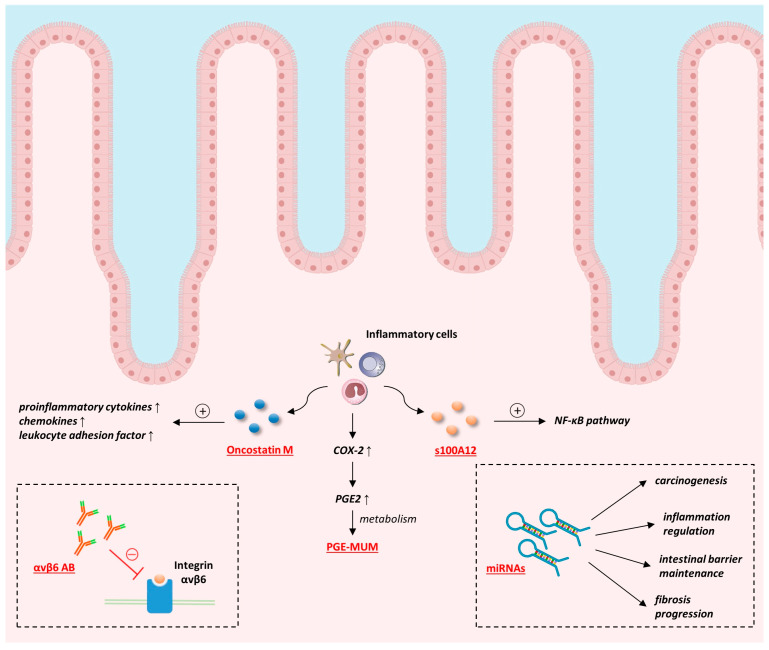
Pathophysiological mechanisms of emerging biomarkers in inflammatory bowel disease. Abbreviations: PGE-MUM—prostaglandin E-major urinary metabolite; miRNA—micro RNA; NF-κB—Nuclear factor kappa-light-chain-enhancer of activated B cells; COX-2—cyclooxygenase-2; PGE2—prostaglandin E2.

S100A12 is a calcium-binding protein (10.4 kDa), also known as calgranulin-c, that shows antimicrobial activity. When stored at room temperature, it may remain stable for up to 10 days [43]. It is secreted by neutrophils present in the inflamed intestinal mucosa and, therefore, demonstrates high expression in IBD patients [43,50]. S100A12 induces the release of inflammatory cytokines (such as TNF-α) due to activation of the nuclear factor-κB signal transduction pathway (Figure 2) [78]. Recent research has shown promising results for S100A12 as a diagnostic biomarker in IBD, particularly in pediatric populations. Witarto et al. recently conducted a meta-analysis that included seven studies (712 children and adolescents) and assessed the diagnostic accuracy of S100A12. They revealed an excellent performance of S100A12 in diagnosing IBD in the pediatric population with a pooled sensitivity of 95%, specificity of 97%, and an AUROC of 0.99 (Table 1) [50].

In adult populations, studies have also shown promising results, albeit with some variability. A recent study investigated the accuracy of fecal S100A12 in distinguishing adult patients with IBD from IBS on a cohort comprised of 171 patients with infective gastroenteritis, CD, UC, or IBS and 24 healthy controls. The marker efficiently distinguished patients with IBD from healthy controls (sensitivity 86%, specificity 100%) and patients with IBS (sensitivity 86%, specificity 96%) [51]. These results suggest that S100A12 could be a valuable tool in differentiating IBD from functional gastrointestinal disorders in adults. However, not all studies have shown such high performance. Another study proposed a cut-off value of 54.4 ng/mL in distinguishing patients with IBD and IBS and found lower performance indices (sensitivity 66.7%, specificity 64.4%, AUROC 0.67). Additionally, they found no statistically significant difference between S100A12 values among patients with active or inactive IBD [52]. This discrepancy highlights the need for further research to establish optimal cut-off values and to understand the relationship between S100A12 levels and disease activity. The usage of S100A12 in the assessment of therapy response was also explored, with mixed results. According to the study published by Boschetti et al., fecal S100A12 is not a reliable marker of early clinical response of patients with CD on anti-TNF treatment since it showed no statistically significant reduction in responders [79]. This finding suggests that while S100A12 may be useful for diagnosis, its role in monitoring treatment response may be limited.

In conclusion, while S100A12 shows promise as a biomarker for IBD, particularly in pediatric populations, its performance in adult populations and its utility in monitoring disease activity and treatment response requires further investigation. Additional clinical studies in adults with IBD are necessary to determine optimal cut-off values for diagnosing IBD and detecting clinically active disease. Future research should also focus on comparing S100A12 with other established biomarkers and exploring its potential in combination with other markers to improve diagnostic and monitoring accuracy in IBD.

## 4. Emerging Biomarkers

### 4.1. αvβ6 Antibody

Integrin αvβ6 is expressed in epithelial cells, where it serves as a receptor for extracellular matrix proteins and is responsible for maintaining the integrity of the epithelial barrier [80,81]. Since the epithelial barrier is damaged in patients with UC, authors hypothesized that antibodies against αvβ6 receptors might be responsible for this impairment [80]. Hence, it has been the subject of investigation to understand its role in disease pathogenesis and as a potential therapeutic target (Figure 2).

Therefore, a study was conducted on a cohort that included 112 patients with UC and 155 controls and screened for 23 integrin proteins using the enzyme-linked immunosorbent assays (ELISA) [80]. They found a very high sensitivity (92%) and specificity (94.8%) of αvβ6 in the diagnosis of UC. Additionally, the level of antibodies correlated well with the severity of UC [80] (Table 2). Moreover, antibody levels correlated well with disease severity, suggesting potential utility in monitoring disease activity. These results have been further tested on a Swedish cohort of adult patients with IBD and IBS [82]. Authors found significantly higher IgG anti-αvβ6 values in patients with UC than in the groups with CD, accompanied by a high diagnostic sensitivity and specificity of 76.3% and 79%, respectively [82]. They also detected significantly higher values in patients with UC when compared with the IBS group, with a diagnostic specificity of 96% [82].

Since the loss of epithelial barrier integrity is an early feature of the disease, American authors hypothesized that anti-αvβ6 antibodies might be present years before the clinical manifestation of the disease [91]. Therefore, they tested the presence of antibodies in preclinical UC phases and found significantly higher values among patients who developed UC compared to controls for up to 10 years before the diagnosis. The preclinical diagnostic performance was excellent, with an area under the receiver operating characteristic curve (AUROC) of 0.8. Furthermore, high levels of anti-αvβ6 antibodies were associated with a more severe disease course, suggesting potential prognostic value [91]. While these results are promising, several important considerations warrant further investigation. Longitudinal studies are still essential to determine if αvβ6 antibody levels fluctuate with disease activity and treatment response, which could potentially make these antibodies a useful monitoring tool. Additionally, the specificity of the antibodies for UC vs. other forms of colitis should be further evaluated to ensure diagnostic accuracy in clinical practice. The mechanistic role of these antibodies in UC pathogenesis remains to be fully elucidated, as understanding whether they are causative or only a consequence of the disease process could inform potential therapeutic strategies. Moreover, the predictive value of the antibodies for disease progression and treatment response should be assessed in larger, prospective cohorts. Finally, standardization of the assay and the establishment of universally accepted cut-off values are necessary for their widespread clinical implementation. In conclusion, anti-integrin αvβ6 antibodies show great promise as a biomarker for UC, potentially offering advantages in diagnosis, prognosis, and disease monitoring. However, further research is needed to fully validate their clinical utility and understand their role in UC pathogenesis before they can be widely adopted in clinical practice.

### 4.2. Prostaglandin E-Major Urinary Metabolite (PGE-MUM)

The final treatment goal in the management of UC is to achieve not only clinical but also endoscopic remission and, ultimately, mucosal healing, which is associated with a reduction of UC recurrence and a lower risk for the development of colorectal cancer [82]. In order to avoid invasive endoscopic monitoring due to patient discomfort and high costs, new biomarkers that reflect the disease activity have emerged. During the inflammatory phase of UC, cytokines cause an upregulation of cyclooxygenase-2 (COX-2) that leads to prostaglandin E2 (PGE2) secretion in colonic mucosa (Figure 2). PGE2 inhibits electrolyte absorption, promotes vascular permeability, and causes hyperperistalsis [91]. It is further metabolized and finally excreted in urine as the prostaglandin E-major urinary metabolite (PGE-MUM) [84]. PGE-MUM is a highly stable molecule that might be measured in urine samples using liquid chromatography, mass spectrometry, and radioimmunoassay methods [84,92].

Ishida et al. evaluated the performance of PGE-MUM in the assessment of UC activity by comparing it with the fecal immunochemical occult blood test (FIT). The authors found a significant correlation between PGE-MUM and FIT, while the disease activity was defined using the endoscopic Mayo score and Ulcerative Colitis Endoscopic Index of Severity (UCEIS) score [93] (Table 2). Another further study assessed the correlation of PGE-MUM and CRP and compared these biomarkers with the endoscopic activity scores. PGE-MUM correlated stronger with endoscopic scores than CRP (r = 0.518, *p* < 0.001 vs. r = 0.444, *p* < 0.001, respectively) [83]. This suggests that PGE-MUM may be a more accurate reflection of mucosal inflammation than the systemic inflammatory marker CRP. Additionally, Arai et al. proposed cut-off values of PGE-MUM in predicting endoscopic (21.8 μg/g·Cr) and histological (17.0 μg/g·Cr) activity with reported sensitivities of 81–82% [84]. These thresholds could potentially be used to guide clinical decision-making, although further validation in diverse patient populations is needed. Finally, a recently published study investigated the potential of PGE-MUM in predicting disease relapse, and the authors proposed a cut-off value of 25.2 mg/g Cr (AUROC 0.721) to determine inflammation recurrence [94]. This predictive capability could be particularly valuable in identifying patients at higher risk of relapse who may benefit from more intensive monitoring or treatment escalation. Apart from reflecting UC activity, the performance of PGE-MUM in diagnosing endoscopic and histological remission of patients with UC has also been evaluated. By evaluating the cohort of 128 patients, the authors found a statistically significant difference in PGE-MUM values between groups with endoscopic/histological/histo-endoscopic remission when compared to the patients with active disease [92]. This suggests that PGE-MUM could potentially be used to assess mucosal healing non-invasively.

However, even though these results show promise, several considerations should be noted. The studies to date have been relatively small and mostly single-center, necessitating larger, multi-center studies to validate these findings and establish widely applicable cut-off values. Further investigation is needed to compare the performance of PGE-MUM relative to other established biomarkers, such as fecal calprotectin. Additionally, the impact of factors such as disease extent, treatment history, and comorbidities on PGE-MUM levels should be explored. Finally, evaluating in detail the cost-effectiveness of PGE-MUM testing compared to other monitoring strategies is also essential. In conclusion, PGE-MUM shows promising performance as a non-invasive biomarker for disease monitoring and relapse prediction in UC patients. Its ability to reflect both endoscopic and histological activity, coupled with the convenience of urine sampling, makes it an attractive option for clinical practice. However, further research is needed to fully establish its role in UC management algorithms and to determine how it can be best integrated with other clinical and laboratory parameters to optimize patient care.

### 4.3. MicroRNA (miRNA)

Micro ribonucleic acids (miRNAs) are single-stranded non-coding RNAs that consist of 18–23 nucleotides and regulate gene expression on a post-transcriptional basis [95,96,97]. Due to their stability and presence in multiple tissues and body secretions, such as intestinal mucosa, blood, feces, and saliva, they represent potential biomarkers and therapeutic targets, particularly in the context of personalized medicine [95]. miRNAs affect various cellular and metabolic pathways, such as cell proliferation, differentiation, signaling, and apoptosis, as well as inflammation and carcinogenesis, but are also considered important immune modulators [98] (Figure 2). miRNAs dysregulation has been observed in patients with IBD, and it is considered they are involved in several pathophysiological processes, such as inflammatory reactions, maintenance of intestinal barrier, and autophagy of intestinal epithelium [99]. Zhang L. et al. revealed a role of miR-21 in the regulation of the intestinal epithelial tight junction permeability via the PTEN/PI3K/Akt signaling pathway. miR-21 downregulation significantly decreased the intestinal permeability but also decreased the levels of proinflammatory cytokines (IL-6, IL-8, and prostaglandin E2) in the cell culture medium [100]. Another miRNA found to induce intestinal inflammation in patients with CD is miR-124. Its upregulation aggravated the experimental colitis via the inhibition of aryl hydrocarbon receptor (AHR), which is proved to be responsible for the lowering of proinflammatory cytokines (IL-6, IL-7, IL-12, IL-17, TNF, and IFN Y), reduction of microbial translocation and development of fibrosis in the colon [99,100,101,102]. miR-122a, miR-191a, miR-212, miR-675 and miR-874 also seem to weaken the intestinal barrier. While the miR-874 decreases aquaporin expression, miR-191a, miR-212, and miR-675 act via zonula occludens (ZO)-1 [99]. Contrary, miR-93 and miR-200b strengthen the intestinal barrier by targeting protein tyrosine kinase 6 (PTK6) and c-JUN, respectively [99].

Studies also confirmed miRNAs may help differentiate IBD from IBS, predict the disease course severity, and prognosticate the development of extraintestinal manifestations [95]. Lately, newer techniques for miRNA detection have emerged and tend to replace traditional methods that include Northern blotting, microarrays, and Quantitative reverse transcription polymerase chain reaction (RT-qPCR) due to their low specificity and sensitivity. Newer techniques include fluorescent in situ hybridization, nucleic acid, enzyme-free amplification, and nanomaterial-based miRNA detection [95].

A recent study conducted in China proved that miRNAs may have a role in the prediction of therapeutic response since they found a significant downregulation of 6 different miRNAs in patients resistant to glucocorticoid therapy. Among detected miRNAs, miR-16-2-3p, miR-150-5p, and miR-224-5p showed the highest specificity (97.3%), and miR-32-5p the highest sensitivity (97.4%) in the prediction of glucocorticoid response [86] (Table 2). Moreover, another recently published study evaluated the presence of serum miRNA-675-5p in IBD patients and found significantly higher values in patients with active UC vs. patients in remission (*p* = 0.02). Additionally, miRNA-675-5p showed excellent performance in distinguishing both UC and CD patients from healthy controls (specificity 97.3% and sensitivity 85.7%; specificity 95.2% and sensitivity 88.4%, respectively) [85].

Due to their high stability, miRNAs arise as an emerging biomarker in the diagnosis and monitoring of IBD patients. These small regulators of gene expression present an important target in the therapeutic armamentarium against inflammatory and fibrosis processes in IBD patients [103]. However, while the potential of miRNAs as biomarkers and therapeutic targets in IBD is promising, several challenges remain. There is a need for standardized protocols for miRNA isolation, quantification, and data analysis to ensure reproducibility across studies. Many miRNAs are involved in multiple pathways, which may limit their specificity as biomarkers or therapeutic targets for IBD. For therapeutic applications, efficient and targeted delivery of miRNA mimics or inhibitors to the intestinal mucosa remains a challenge. Additionally, large-scale, prospective studies are needed to validate the diagnostic and prognostic value of miRNA biomarkers in diverse patient populations. In conclusion, miRNAs represent a promising avenue for improving IBD diagnosis, monitoring, and treatment. Their stability and presence in various biological samples make them attractive biomarkers, while their role in regulating gene expression offers potential therapeutic applications. However, while miRNAs show great promise as biomarkers for IBD, there are still significant challenges to overcome before they can be widely adopted into regular clinical practice. One major hurdle is the high cost associated with miRNA detection and analysis. Current methods for miRNA profiling, such as next-generation sequencing and microarrays, require expensive equipment and reagents. Additionally, the complexity of data analysis often necessitates specialized bioinformatics expertise, further increasing costs. These financial barriers can limit the feasibility of large-scale studies needed to validate miRNA biomarkers and may hinder their implementation in routine clinical care, especially in resource-limited settings. Furthermore, the lack of standardized protocols for miRNA isolation, quantification, and data analysis contributes to variability between studies and increases the overall cost of research. Developing more cost-effective and standardized methods for miRNA detection and analysis will be crucial for translating these promising biomarkers into practical clinical tools. Despite these challenges, ongoing technological advancements and decreasing costs of molecular techniques suggest that miRNA-based diagnostics and prognostics may become more accessible in the future, potentially revolutionizing personalized medicine approaches in IBD management.

### 4.4. Oncostatin M

Oncostatin M (OSM) is a cytokine belonging to the interleukin 6 (IL-6) family, produced by T cells, monocytes, macrophages, dendritic cells, and activated neutrophils that may swiftly produce high amounts of OSM after the stimulation [87,104]. Like the rest of the IL-6 family, it is a proinflammatory cytokine with the potential to activate endothelial and stromal cells and promote leukocyte recruitment [104]. It is also an important factor in the process of fibrogenesis [104] (Figure 2). In 2017, West et al. discovered that patients with IBD express high levels of both OSM and its receptor (OSMR) [105]. OSM receptor binding stimulates the release of proinflammatory cytokines, chemokines, and leukocyte adhesion factor [105,106]. High OSM expression was found in the tissues and blood of IBD patients, and its concentration is related to the severity of the disease [87,106]. One of the most promising aspects of OSM research in IBD is its potential as a predictor of treatment response. Several studies proved that high expression of OSM before the treatment initiation was strongly associated with the failure of anti-TNF therapy [87,106]. This could present the cornerstone of personalized treatment due to the significant rate of patients with primary non-response or secondary loss of response [106].

However, OSM in serum showed low specificity due to high expression in other pathologic conditions such as sepsis or spondylarthritis, dermatitis, gingivitis, and carcinogenesis [87,88]. After the initial studies that evaluated OSM in serum and tissues, the apparent need for non-invasive and more practical OSM testing became clear. Therefore, Cao et al. tested the efficacy of fecal OSM alone and in combination with fecal calprotectin on three levels: diagnosing IBD, testing disease activity, and predicting response on infliximab therapy [87]. They found a positive correlation between OSM and both endoscopic and clinical disease activity. Expectedly, the best performance values in diagnosing IBD and predicting therapeutic response at week 28 were detected when using the combination of these biomarkers, with AUROCs of 0.93 and 0.859, respectively. However, it showed no benefit in disease activity monitoring [87] (Table 2).

Furthermore, another study evaluated the efficacy of OSM in the assessment of disease activity and prediction of infliximab response in patients with IBD using the chemiluminescence immunoassay to measure serum OSM levels. They proposed cut-off values for identification of mucosal healing (64.1 pg/mL, AUROC 0.84), clinical response (83 pg/mL, AUROC 0.90), and clinical remission (98.9 pg/mL, AUROC 0.9) with reported sensitivities and specificities ranging from 80 to 90% [88,107]. Also, Bertani et al. evaluated the efficacy of OSM in the prediction of mucosal healing at week 54 in patients treated with anti-TNF or vedolizumab. The authors found a significant association of low OSM levels with mucosal healing in the anti-TNF group with a diagnostic accuracy of 0.91, but not in the vedolizumab group (AUROC 0.56) [106]. Other studies also found inadequate performance of OSM in predicting response to vedolizumab or ustekinumab therapy [88,105,108].

Recently, a meta-analysis that included 16 studies (818 patients with CD and 686 with UC treated with anti-TNF) was conducted in order to determine OSM association with the IBD severity [88]. They found significant correlations between OSM and endoscopic scores, fecal calprotectin, and CRP. Meta-analysis also revealed significantly higher OSM levels in non-responders than responders, non-remitters than remitters, and in patients without mucosal healing compared to those with mucosal healing [88]. While neutralizing OSM antibodies is being developed, OSM has a high potential to predict outcomes of anti-TNF therapy in IBD patients [87].

While these findings are promising, several limitations and challenges remain. The specificity of serum OSM as a biomarker needs improvement, given its elevation in various inflammatory conditions. Additionally, the optimal method for measuring OSM (serum vs. fecal vs. tissue) still needs to be established. Standardization of OSM measurement techniques and cut-off values across different clinical settings is also necessary. Further investigation is required to determine the role of OSM in predicting response to non-anti-TNF biologics. Moreover, the potential of OSM as a therapeutic target needs to be explored, with neutralizing OSM antibodies currently in development. Hence, given all the information, OSM shows great promise as a biomarker for predicting outcomes of anti-TNF therapy in IBD patients. However, further research is needed to overcome current limitations and to fully elucidate its role in IBD pathogenesis and management. As neutralizing OSM antibodies are being developed, OSM may also emerge as a potential therapeutic target in the future.

### 4.5. B-Cell Activating Factor (BAFF)

B-cell Activating Factor (BAFF) is a cytokine belonging to the tumor necrosis factor (TNF) superfamily, produced by myeloid cells such as monocytes, macrophages, dendritic cells, and neutrophils [109]. BAFF plays a crucial role in immune cell development and function, primarily targeting B-cells. It promotes B-cell survival, maturation, and function by regulating apoptotic molecules [110]. BAFF also supports high-affinity B-cell clones and class switch recombination and can lead to B-cell expansion and autoantibody production in overexpression scenarios. Additionally, BAFF co-stimulates T-cell activation and differentiation and activates monocytes and dendritic cells [111,112]. Elevated BAFF levels are observed in autoimmune diseases like SLE, rheumatoid arthritis, and Sjogren’s syndrome, correlating with higher autoantibody levels and disease activity [113,114,115]. Moreover, BAFF inhibitors, such as belimumab, are even approved for SLE treatment [113]. Additionally, BAFF may contribute to B-cell malignancies like non-Hodgkin’s lymphoma, chronic lymphocytic leukemia, and multiple myeloma through abnormal expression and production [116,117,118].

In IBD patients, BAFF expression is linked to inflammation, with high levels in the intestinal mucosa. This overexpression activates the NF-κB signaling pathway and the NLRP3 inflammasome, key players in the inflammatory response [89,119]. In the further context of IBD, BAFF has emerged as a potentially significant biomarker, as studies have shown that patients with IBD, including both CD and UC, exhibit elevated levels of BAFF in serum, feces, and colonic tissues [89,120]. Furthermore, Zhang et al. found that fecal BAFF concentrations were significantly higher in IBD patients compared to IBS patients and healthy controls, with a cut-off value of 325 pg/mL showing high sensitivity (90%) for distinguishing active IBD (Table 2). Serum BAFF had similar specificity (93%) but lower sensitivity (55%). Moreover, the concentration of BAFF has been shown to correlate with disease activity, making it a potential marker for IBD monitoring [89]. Fu et al. compared fecal BAFF, calprotectin, and fecal occult blood test (FOBT) for distinguishing IBD from IBS [90]. Fecal BAFF ≥ 227.3 pg/mL showed 84% sensitivity and 100% specificity, calprotectin ≥ 50 µg/g had 76% sensitivity and 93% specificity, and FOBT had 65% sensitivity and 93% specificity. Combining BAFF with calprotectin increased accuracy to 94% sensitivity and 93% specificity. Fecal BAFF also correlated more strongly with endoscopic inflammatory scores than calprotectin in UC and CD [90]. Fodor et al. found higher fecal BAFF in IBD compared to IBS and healthy groups, with pediatric UC patients showing higher levels than CD patients. Fecal BAFF had moderate sensitivity (51%) and high specificity (93%) for distinguishing IBD from IBS in children [121]. These studies suggest that BAFF, particularly fecal BAFF, could be a valuable biomarker for diagnosing and monitoring IBD. However, more extensive studies are needed to validate these findings and establish standardized cut-off values.

Several studies have evaluated the role of BAFF in predicting treatment response. For instance, elevated serum BAFF levels at baseline have been associated with a better response to infliximab (IFX) treatment in CD patients. Responders to IFX treatment showed a reduction in BAFF levels post-treatment, while non-responders exhibited an increase [122,123]. Additionally, specific single nucleotide polymorphisms (SNPs) in the BAFF gene, such as rs1041569, have been linked to CD susceptibility and treatment response [122]. Recent studies have also highlighted the potential of BAFF blockade as a therapeutic strategy. In experimental models, BAFF blockade has been shown to improve inflammatory status, reduce body weight loss, and decrease histopathological damage in colitis [124,125,126]. This suggests that targeting BAFF could be a viable approach for managing IBD.

According to all presented data, it is possible that BAFF will play a future role as a biomarker and therapeutic target that offers new avenues for personalized treatment strategies in IBD patients [120]. However, several challenges and considerations still remain. BAFF elevation is not specific to IBD and occurs in other inflammatory conditions, necessitating more research to determine its specificity in various clinical scenarios. Standardized methods for measuring BAFF and established cut-off values are necessary for clinical implementation. Although BAFF blockade has shown promise in experimental models, clinical trials are needed to evaluate the efficacy and safety of BAFF-targeted therapies in IBD patients. Given BAFF’s role in normal B cell function, careful consideration must be given to the potential adverse effects of BAFF inhibition on protective immunity. Additionally, the potential of combining BAFF inhibition with other targeted therapies in IBD should be explored. In conclusion, BAFF represents a promising avenue for both biomarker development and targeted therapy in IBD. Its ability to reflect disease activity and its potential role in pathogenesis make it an attractive subject for further research. However, more studies are needed to fully elucidate its role in IBD and translate these findings into clinical practice. The development of BAFF-targeted therapies could potentially offer new personalized treatment strategies for IBD patients, but this approach requires careful evaluation in clinical trials.

## 5. Other Biomarkers with Potential Usefulness in IBD Management

In addition to the mentioned biomarkers that could enhance comprehensive IBD management, several more warrant consideration for their potential utility in IBD management and patient care, provided they are correctly interpreted.

Albumin is one such biomarker, despite some limitations that necessitate careful interpretation alongside other clinical indices. Studies have shown that low serum albumin levels are associated with active inflammation and malnutrition in IBD patients, reflecting both nutritional status and disease activity, which makes it a non-specific marker [127]. However, as Khan et al. demonstrated in a 2017 study, hypoalbuminemia correlates with increased disease severity, risk of complications, and the need for surgery in IBD patients. Low albumin levels at diagnosis can predict a more severe disease course and an increased risk of relapse [127,128]. Its limitations include the inability to serve as a definitive prognostic marker of malnutrition in IBD due to its dual reflection of inflammation and nutrition. Additionally, its relatively long half-life (19–21 days) makes it less responsive to acute changes compared to other markers [129]. Nevertheless, albumin’s utility improves when combined with other markers, such as the C-reactive protein to albumin ratio (CAR), which has shown promise as a more accurate marker of disease activity than albumin alone. Combining albumin with other biomarkers or clinical parameters may enhance its predictive value [130,131]. While not perfect, serum albumin remains a widely available and relatively inexpensive biomarker that can provide valuable information when interpreted within the context of other clinical and laboratory findings. However, clinicians should be aware of its non-specific nature and interpret albumin levels in the context of the overall clinical picture.

Fibrinogen levels are significantly increased in patients with active IBD compared to those in remission or healthy controls [132]. Elevated fibrinogen levels are also associated with both UC and CD and correlate positively with clinical disease activity scores [132,133]. Fibrinogen can independently distinguish active disease from remission in both UC and CD, with AUROC values of 0.806 for UC and 0.869 for CD, demonstrating higher discriminative capacity for active IBD compared to markers like red cell distribution width (RDW), ESR, neutrophil-to-lymphocyte ratio (NLR), and platelet-to-lymphocyte ratio (PLR) [132]. However, C-reactive protein (CRP) performs better than fibrinogen in identifying active IBD [132]. Fibrinogen also contributes to a hypercoagulable state and increased thromboembolism risk, particularly in UC patients [133]. Despite its utility, fibrinogen alone is not a definitive marker for IBD activity or mucosal healing and should be used with other clinical and laboratory findings for optimal disease assessment [22]. Combining fibrinogen with other biomarkers or clinical parameters may enhance its predictive value in IBD management. Further research is needed to fully understand its role in predicting treatment response and long-term outcomes.

Serum amyloid A (SAA) is an acute-phase protein synthesized primarily by the liver in response to inflammatory stimuli. As an apolipoprotein of high-density lipoproteins (HDL), SAA belongs to the family of acute-phase reactants [134]. Several studies suggest that SAA may be a better biomarker of disease activity in IBD compared to CRP, showing a strong correlation with mucosal inflammation and the ability to predict a lack of mucosal healing in IBD patients [134,135]. SAA levels significantly increase in both UC and CD patients with active disease compared to those in remission, demonstrating good discriminative capacity for identifying active IBD, with an area under the ROC curve value of 0.81 reported in some studies [135]. SAA may be particularly useful in patients who do not have elevated CRP levels despite having active disease. It correlates well with other inflammatory markers like fecal calprotectin, IL-6, and endoscopic scores of disease activity [134,135,136]. Additionally, SAA can stimulate protective and anti-inflammatory IL-22-producing neutrophils, potentially protecting the epithelial barrier [136]. While promising, SAA alone is not considered a definitive marker for IBD activity or mucosal healing, as its levels can be affected by other inflammatory conditions, being a general acute phase reactant [137]. Combining SAA with other biomarkers or clinical parameters may enhance its predictive value in IBD management. Further research is needed to clarify its role in predicting treatment response and long-term outcomes. SAA shows promise as a biomarker for assessing disease activity, predicting mucosal healing, and identifying active inflammation even when CRP levels are normal. However, it should be used as part of a comprehensive assessment rather than as a standalone marker.

Globulin plays a crucial role in immunity and inflammation, with increased levels associated with the progression of IBD [138]. An elevated serum globulin fraction is independently linked to greater disease severity in IBD patients and may serve as a biomarker of disease severity over several years [138,139]. Patients with high globulin fractions experience increased healthcare utilization, including more emergency department visits, hospitalizations, and IBD-related surgeries [139]. In ulcerative colitis, serum globulin levels are significantly positively correlated with endoscopic activity [140]. The albumin-to-globulin ratio (AGR) has been studied as a potential marker of inflammatory disease in IBD, with the globulin fraction providing additional information beyond traditional markers [138]. More research is needed to fully establish the role of the serum globulin fraction in IBD management. It should be used in conjunction with other clinical and laboratory findings for optimal disease assessment, while its ability to provide information over extended periods makes it of potential value for long-term disease monitoring [139]. Further studies are necessary to fully elucidate its role in clinical practice and determine how best to integrate it with other established biomarkers in IBD management.

α1-Acid Glycoprotein (AGP) is an acute phase protein synthesized primarily by the liver in response to inflammatory stimuli, playing roles in immune modulation, drug binding and transport, and maintaining capillary barrier function [141]. AGP levels are significantly increased in patients with active IBD compared to those in remission or healthy controls, correlating with clinical disease activity in both UC and CD. High AGP levels have prognostic value for an increased risk of relapse in IBD [142]. AGP is considered a slower acute phase reactant, making it less responsive to acute changes compared to markers like CRP. However, it may be useful in assessing disease activity in IBD [143]. AGP alone is not a definitive marker for IBD activity or mucosal healing, as its levels can be affected by other inflammatory conditions [144]. Combining AGP with other biomarkers or clinical parameters may improve its predictive value in IBD management. Advances in proteomics and metabolomics may enhance understanding of AGP’s role in IBD pathogenesis and its potential as a biomarker. However, it should be part of a comprehensive assessment rather than a standalone marker. Further research is needed to fully elucidate its role in IBD pathogenesis and optimize its use in clinical practice.

Finally, as proposed by Nowak et al., promising biomarkers for assessing UC risk include stool proteolytic activity, potentially augmented by a polygenic risk score [14]. Additionally, for UC diagnosis, a combination of anti-αvβ6 antibodies, PR3-ANCA levels, serum OSM, and serum CPa9-HNE could be effective. They also suggest several other biomarkers that can be of use in disease management that are under development, such as serum TFF3, bile acids, CPa9-HNE, and gelsolin [14].

## 6. Current Trends in Biomarkers Research

Biomarker, as a non-invasive and reproducible tool, plays an increasingly important role in the management of patients with IBD. Certain biomarkers, such as primarily CRP and fecal calprotectin, have become indispensable in the management of IBD patients. However, the need for new biomarkers is increasingly becoming the subject of numerous studies. Current trends in the discovery of new biomarkers in the management of IBD patients can mainly be found in the domains of proteomics, genetics, and metabolomics [27,145]. As is known, there is no single, unique biomarker that would be sufficient in all phases of the management of IBD patients. Technological advancements contribute to the identification of an individual panel of biomarkers, which further personalizes the approach of patients suffering from IBD. However, challenges remain in implementing personalized medicine in routine clinical practice. Integration of diverse biomarkers, standardization of assays, and data interpretation pose logistical and analytical challenges. Furthermore, ethical considerations, data privacy concerns, and healthcare disparities necessitate careful navigation through the concept of personalized medicine.

### 6.1. Proteomics

Proteomics not only represents a study of the set of gene-encoded proteins known as the proteome but also includes the study of proteins’ isoforms, post-translational modifications, and protein-protein interactions [145]. Due to the strong bond between protein expression and disease activity, the application of proteomics in biomarker discovery is promising regarding new findings in IBD pathogenesis, as well as in revealing novel biomarkers. The improvement of molecular technology significantly contributes to accelerated research in the field of proteomics. The most widely used proteomic technique in IBD research is liquid chromatography coupled with electrospray tandem mass spectrometry (LC–ESI-MS/MS) [27]. Other used techniques are two-dimensional gel electrophoresis coupled with matrix-assisted laser desorption/ionization (MALDI)-MS screening and immunofluorescence microscopy [27].

Proteomics is finding its place in differencing IBD and non-IBD intestinal disease, distinguishing UC and CD, pathogenesis, disease behavior, and prediction of treatment response. One of the most important possible uses of proteomics may be in the prediction of neoplastic transformation [145]. Hence, Brentnall TA et al. performed a study on a small population of UC patients who were divided based on the presence of dysplastic changes or cancer into progressor and non-progressor groups [146]. Authors concluded that the overall protein profile in the non-dysplastic tissue of patients with UC and progressors is closer to dysplastic tissue than to the mucosa of non-progressors, suggesting that there are early changes in the protein expression before the development of histologically visible dysplasia, potentially offering an early warning system for cancer risk in IBD patients [146]. Similar findings have been found in several other studies [147,148], reinforcing the potential of proteomics in cancer risk assessment for IBD patients. However, it is crucial to note that these findings are based on small-scale studies, and larger population-based investigations are necessary to validate and refine these observations. While proteomics shows great promise in IBD research and clinical applications, several challenges remain. These include the standardization of proteomic techniques and data analysis methods, as well as the integration of proteomic data with other omics data (e.g., genomics, transcriptomics, metabolomics). Translating proteomic findings into clinically applicable tools is essential, but the high cost and technical expertise required for large-scale proteomic studies present significant barriers. Despite these challenges, the potential of proteomics to revolutionize our understanding of IBD pathogenesis and improve patient care through personalized medicine approaches remains significant. Future research should focus on validating proteomic biomarkers in large, diverse patient cohorts and developing streamlined, cost-effective proteomic assays for clinical use.

### 6.2. Genetics

Multiple studies suggest that a genetic risk factor for developing inflammatory bowel disease probably modifies the immune response to the intestinal microbiota [27]. Genome-wide association studies (GWASs) have identified approximately 240 gene loci associated with susceptibility to IBD [149]. These researches are helpful not only in understanding disease pathogenesis but also in encouraging the discovery of new biomarkers. Using genetic profiling of blood samples and mucosal biopsies, authors have been able to identify gene panels that distinguish IBD and healthy controls [150]. Several studies concluded that patients with UC and CD have different gene panels [151,152,153]. Furthermore, Burkoff et al. managed to reveal gene profiles in patients with active Crohn’s disease as opposed to patients with CD in remission [154]. The importance of genetic research lies in the fact that a positive family history represents one of the strongest risk factors for the development of IBD, which may affect the phenotype of IBD [155,156]. As some authors emphasize, a positive family history is reported to be a risk factor for developing disease in around 8–12% of IBD patients [157].

In a 34-year cohort study in the Danish population, authors showed that the highest incidence rate of IBD occurred in first-degree relatives (almost eight times higher for CD and four times for UC), second-third-degree relatives, with the highest risk observed early in life (especially <20 years of age) This suggests a potential role for early genetic screening in high-risk populations [158]. Recently, discoveries in IBD genetics have provided possible explanations for the therapy response. A result of the meta-analysis suggested that carrying NOD2 mutations in CD may require aggressive therapeutic strategies, such as anti-TNF therapy [157,159]. This finding highlights the potential for genetic profiling to guide personalized treatment approaches. Furthermore, Nie K et al. conducted a study in which Gene Expression Omnibus (GEO) microarray cohorts with different anti-TNFα responses in patients with CD were screened [160]. In total, four discovered datasets were investigated, with differentially expressed genes between anti-TNFα responders and nonresponders confirmed in each cohort. In the study, bioinformatics and experimental research identified TLR2, TREM1, CXCR1, FPR1, and FPR2 as promising candidates for predicting the anti-TNFα response in patients with Crohn’s disease and especially TLR2 as a core predictor [160]. These findings could potentially lead to the development of genetic tests to predict treatment response, allowing for more targeted and effective therapy.

Moreover, efforts have been made to detect genetic polymorphisms with the aim of predicting early response to anti-TNF drugs in inflammatory bowel disease. However, it would be of considerable clinical interest to identify and validate genetic biomarkers of long-term response. A study conducted in Spain, analyzing the usefulness of biomarkers of response to anti-TNFs in pediatric IBD as long-term biomarkers, showed DNA variants specific to disease type and anti-TNF type in the pediatric population [161]. The study included 340 children diagnosed with IBD who were treated with infliximab or adalimumab. Using real-time PCR, authors showed that variants C rs10508884 (CXCL12), A rs2241880 (ATG16L1), and T rs6100556 (PHACTR3) (*p*-value 0.049; *p*-value 0.03; *p*-value 0.031, respectively) were associated with worse long-term response to anti-TNFs in pediatric IBD patients. The authors concluded that genotyping these genetic variants before initiation of anti-TNFs would enable the identification of pediatric patients who are long-term responders to this therapy [161]. Of course, these findings should be further validated in prospective studies.

While these genetic discoveries hold great promise for personalized medicine in IBD, some challenges still remain. The complex interplay between multiple genetic loci and environmental factors in IBD pathogenesis makes it difficult to translate genetic findings directly into clinical practice. Moreover, the predictive value of individual genetic markers is often limited, necessitating the development of more comprehensive genetic risk scores. Future research should focus on validating these genetic biomarkers in larger, diverse patient cohorts and prospective studies. Integration of genetic data with other molecular and clinical information may provide a more comprehensive approach to predicting disease course and treatment response. Finally, genetic research in IBD has made significant strides in recent years, offering new insights into disease pathogenesis and potential avenues for personalized medicine. However, translating these genetic discoveries into clinically useful tools for diagnosis, prognosis, and treatment selection remains a key challenge for the field. Continued research and validation studies are necessary to fully harness the potential of genetic biomarkers in IBD management.

### 6.3. Epigenetics

Epigenetics represents the study of mitotically heritable changes in the genome function without a change in nucleotide sequence [162]. Epigenetic mechanisms that can explain changes in gene function caused by gene-environment interactions rather than changes in the DNA sequence itself include DNA methylation, histone acetylation, RNA interference, and the positioning of nucleosomes [27,162]. Studies have shown the importance of DNA methylation in the pathogenesis of IBD, suggesting a possible role of modified genes as biomarkers [163,164]. Cooke et al. conducted a study with genome-wide methylation profiling using the HumanMethylation27 BeadChip microarray on rectal mucosa specimens. Results showed evidence of differential methylation in CD and UC specimens in comparison to those from healthy controls [164]. Gene showing significant evidence of differential methylation in both ulcerative colitis and Crohn’s disease included THRAP2, FANCC, GBGT1, DOK2, TNFSF4, TNFSF12, and FUT7. Authors suggested that consistent differences in DNA methylation between IBD cases and controls at regulatory sites within these genes suggest that their altered transcription contributes to IBD pathogenesis [164].

Epigenetics may be the major reason for determining the IBD outcome of colorectal cancer due to the obvious connection between epigenetics, cell division, and cancer [165]. The link between epigenetic alterations, cell division, and cancer development suggests that epigenetic changes may be a major factor in determining CRC risk in IBD patients. As group of authors suggested, linoleic acid and 12 hydroxy 8,10-octadecadienoic acid, serum M2-pyruvate kinase and six metabolic genes (NAT2, XDH, GPX3, AKR1C4, SPHK1, and ADCY5) expression represent possible biomarkers for early detection and transition to CRC condition, emphasizing the significance of metabolism reprogramming in IBD and CRC [165]. These findings highlight the importance of metabolic reprogramming in the progression from IBD to CRC.

Peripheral blood mononuclear cells’ TFPI2 and NDRG4 gene promoter methylation analyses are emerging as novel, non-invasive epigenetic biomarkers that can be utilized as prognostic indicators in IBD. Additionally, the importance of microRNA (miRNA) profiling in linking colon inflammation to colorectal cancer (CRC) has been underscored. Specifically, miR-31, miR-139-5p, miR-155, miR-17, miR-223, miR-370-3p, miR-106a, miR-135b, and miR-320 have been identified as potential biomarkers to estimate the risk of IBD progressing to colorectal cancer [165].

However, even though we have promising results in the field of epigenetics, several challenges remain in translating research into clinical practice. Epigenetic patterns can vary significantly between different cell types within the same tissue, making it challenging to interpret results from bulk tissue samples. Also, determining whether observed epigenetic changes are causal factors in IBD pathogenesis or consequences of the disease process remains difficult. Epigenetic modifications can change over time and in response to environmental factors, necessitating longitudinal studies to fully understand their role in IBD progression. Current methods for epigenetic profiling can be costly and time-consuming, limiting their widespread clinical application, while establishing the functional consequences of observed epigenetic changes requires extensive experimental work. Future research should focus on addressing these challenges by utilizing single-cell epigenomic profiling to account for cellular heterogeneity, integrating epigenetic data with genetic, transcriptomic, and proteomic information for a more comprehensive understanding of IBD pathogenesis, and conducting longitudinal studies to track epigenetic changes over the course of disease progression and treatment. Additionally, there is a need to develop more cost-effective and high-throughput epigenetic profiling methods, as well as to perform functional studies to elucidate the mechanistic roles of identified epigenetic alterations in IBD. Considering all the given information, epigenetics represents a promising field for advancing our understanding of IBD pathogenesis and identifying novel biomarkers for disease progression and treatment response. However, further research is still needed to overcome current limitations and translate epigenetic findings into clinically useful tools.

### 6.4. Metabolomics and Gut Microbiota

Metabolomics implies the study of the complete expression and biological function of small molecules (less than 25 kD) within the biological system [166,167]. However, the metabolite changes refer to both dysbiosis and its affection for the host’s immune response and metabolism [168]. Research in the field of metabolome gives the opportunity to identify biomarkers that reflect the disease activity [168]. In a 2007 study, authors used 1H NMR spectroscopy to examine fecal extracts from IBD patients and healthy controls [169]. It was concluded that metabolic differences in fecal profiles were more marked in the CD group in comparison with the control group, indicating that the inflammation caused by CD is more extensive in comparison with UC and involves the whole intestine [169]. This research is considered pivotal in the field of metabolome research.

Furthermore, Vich Vila et al. approached the investigation of fecal metabolome and its determinants in inflammatory bowel disease to determine the relationship between metabolites and each participant’s lifestyle, clinical characteristics, and gut microbiota composition [170]. They measured 1684 different fecal metabolites and 8 short-chain and branched-chain fatty acids in stool samples of 424 patients with IBD and 255 non-IBD controls. Authors identified over 300 molecules that were differentially abundant in the feces of patients with IBD, especially highlighting the ratio between a sphingolipid and L-urobilin that could discriminate between IBD and non-IBD samples (AUC = 0.85). By identifying alterations in the metabolome of patients with IBD that are independent of diet and surgical history, the authors of this large-scale study shed new light on the interaction between gut microbiota and fecal metabolome [170].

We should emphasize the importance of identifying potential markers for the transition of IBD to CRC. According to researchers, the microbiota plays a crucial role in this transition through the activation of NF-kB and STAT3 signaling pathways. These pathways are often triggered in cancer by various aberrant changes, including epigenetic modifications [171,172]. NF-kB and STAT3 signals contribute to the microenvironment carcinogenesis via inducing pro-inflammatory cytokines production, and these inflammatory mediators upregulate the expression of antiapoptotic genes, cell proliferation, and angiogenesis [172]. Also, in recent studies, serum M2-pyruvate kinase emerged as a promising non-invasive biomarker for colorectal cancer, being promoted as a potential screening test for CRC [173].

Furthermore, Ning L et al. conducted an analysis of 9 metagenomic and 4 metabolomics cohorts of IBD from different populations to evaluate the association of gut microbiota and metabolites with the progression of IBD [174]. Through cross-cohort integrative analysis (CCIA), they revealed consistent characteristics of commensal gut microbiota, especially *Asaccharobacter celatus*, *Gemmiger formicilis, and Erysipelatoclostridium ramosum*, which have been rarely reported in IBD. This study identified 157 microbial species and 206 metabolites that were consistently altered across multiple cohorts, providing a comprehensive view of the IBD-associated microbiome and metabolome. Authors constructed multi-omics biological correlation (MOBC) maps, which highlight gut microbial biotransformation deficiencies and significant alterations in aminoacyl-tRNA synthetases, identifying multi-omics biomarkers for IBD diagnosis, validated across multiple global cohorts (AUROC values ranging from 0.92 to 0.98) [174]. These findings demonstrate the potential of integrating metabolomics and gut microbiota analysis for developing novel diagnostic methods and personalized medicine strategies in IBD management. Furthermore, MOBC maps revealed intricate relationships between microbial species and metabolites, shedding light on potential mechanisms underlying IBD pathogenesis. Notably, the study found that certain microbial species, such as *Bacteroides uniformis* and *Bacteroides vulgatus*, were positively correlated with anti-inflammatory metabolites, while others, like Escherichia coli, showed positive correlations with pro-inflammatory compounds. Additionally, the study revealed that essential genes of the two-component system pathway, linked to fecal calprotectin, are implicated in IBD. This observation provides further insight into the molecular mechanisms underlying the disease and suggests new avenues for therapeutic development. The identification of 36 metabolites with significant differences in IBD patients, whose roles are still largely unknown, opens up opportunities for future research to elucidate their functions and potential as biomarkers or therapeutic targets [174].

While these studies highlight the potential of metabolomics in IBD research and clinical applications, several challenges remain. These include standardizing metabolomic techniques and data analysis, integrating metabolomic data with other omics, validating biomarkers in large, diverse cohorts, and translating findings into clinical tools. Additionally, understanding the functional roles of metabolites in IBD is crucial. Future research should address these challenges to fully harness metabolomics for improving IBD diagnosis, monitoring, and treatment. Longitudinal studies tracking metabolomic changes during disease progression and treatment could provide valuable insights for personalized medicine in IBD management.

### 6.5. Artificial Intelligence and Machine Learning

Recent advancements in biomarker research, alongside the integration of artificial intelligence (AI) and machine learning (ML) technologies, have shown promise in enhancing the precision and efficiency of IBD management [175]. AI and ML technologies have expanded the IBD research field by enabling the analysis of large, complex datasets. These technologies can integrate data from various sources, including genomic, proteomic, transcriptomic, and imaging data, to identify meaningful patterns and associations not apparent through traditional methods. This integration helps further understand the complex etiology of IBD and develop targeted therapies [175,176,177]. The applications of AI in IBD management are diverse and far-reaching. In diagnosis, AI algorithms show high accuracy in distinguishing IBD from other gastrointestinal disorders, reducing the need for invasive procedures. For disease monitoring, AI-powered tools analyze patient-reported symptoms, biomarkers, and imaging results to provide real-time insights into disease activity and predict flare-ups, enabling proactive and personalized management [175]. Machine learning models predict treatment responses by integrating genetic markers, microbiome data, and clinical history, optimizing therapy selection and improving outcomes [175]. In prognosis, AI algorithms forecast long-term disease outcomes and complications by analyzing historical patient data, aiding early intervention and prevention strategies. AI also advances cancer surveillance by detecting subtle mucosal changes indicative of early-stage colorectal cancer, crucial for high-risk IBD patients [175]. In research, AI enhances data analysis through natural language processing of clinical notes, facilitating comprehensive insights into patient populations and disease characteristics [175].

Furthermore, AI technologies have shown great promise in improving the accuracy of endoscopic assessments, which are crucial for diagnosing and monitoring IBD. It is possible that AI can enhance the efficiency and accuracy of assessing baseline endoscopic appearances and the impact of therapeutic interventions on mucosal healing [178]. Additionally, AI systems have the potential to standardize mucosal healing assessments, reducing interobserver variability and improving the reproducibility of endoscopic diagnoses [179]. Also, AI-driven models could be used to predict endoscopic and histologic remission in ulcerative colitis patients using endoscopy data, enhancing the management of the disease [180]. Furthermore, convolutional neural networks (CNNs) have been used to analyze endoscopic images, improving lesion detection and disease severity assessment. Deep learning models based on CNNs can distinguish between UC and CD from colonoscopy images, showing superior accuracy compared to experienced endoscopists in identifying disease types and assessing lesion severity [181]. However, Liu et al. highlighted significant challenges in the current use of AI for image-based prediction in IBD, particularly concerning the risk of bias. They call for improved methodologies and standards to ensure AI models can be reliably used in clinical settings for better patient outcomes [182].

AI models could be used to identify novel biomarkers for IBD, aiding in the prediction of disease progression and treatment responses. These models can integrate various types of data, such as imaging and clinical parameters, to provide comprehensive insights into disease activity [176]. AI-driven models can combine biomarkers, patient-reported outcomes, and disease scores to create predictive models that optimize shared decision-making processes between clinicians and patients [183]. AI and ML could further facilitate precision medicine by enabling the identification of disease subtypes, predicting disease progression, and selecting personalized treatments, thus tailoring treatments to individual patients, improving outcomes, and reducing unnecessary interventions [183].

ML models, such as support vector machines (SVMs) and random forests, have been employed to predict the disease course and response to therapy in IBD patients. These models analyze clinical data, genetic profiles, and biomarker levels to generate personalized treatment recommendations [184,185]. Cai et al. showed that among various ML models, SVM was most effective in predicting disease activity in the CD group, with an AUC of 0.975, sensitivity of 0.947, specificity of 0.920, and accuracy of 0.933 [184]. Furthermore, Derakhshan Nazari et al. developed a statistical method to uncover hidden pathogenic signals in UCN patients, identifying IL6, INHBA, and KRAS as key factors in anti-TNF treatment failure. Additionally, they created a predictive tool using a five-mRNA biomarker panel to forecast anti-TNF mAb response in colitis patients for potential clinical use [186]. While AI and ML have shown great potential in IBD research, their integration into clinical practice still warrants robust prospective validation studies. These studies should involve large, diverse patient cohorts to validate the predictive accuracy and clinical utility of AI models [187,188]. Collaborative efforts between clinicians, data scientists, and bioinformaticians are necessary to translate these technological advancements into practical clinical tools that improve patient outcomes [187,188].

One significant challenge in applying AI to IBD research is the quality and standardization of data. AI models require large, high-quality datasets to generate accurate predictions. However, data heterogeneity and the lack of standardized methodologies can hinder the generalizability of these models [189]. Future efforts should focus on developing standardized data collection and processing protocols to ensure the reliability of AI-driven insights [176,189]. Finally, the use of AI in healthcare raises ethical concerns related to data privacy and security. Ensuring the responsible and transparent use of AI is crucial to maintaining patient trust. Additionally, the interpretability of AI models is a significant concern, as complex algorithms can be challenging to understand and explain. Developing explainable AI models that provide clear insights into their decision-making processes is essential for their adoption in clinical practice [190,191,192].

## 7. Conclusions

The pursuit of effective diagnostic tools for managing patients with IBD has been a focal point of extensive research efforts. While our current arsenal of markers, notably CRP and fecal calprotectin, remains indispensable in clinical practice, they are not without limitations. However, in the still inevitable combination with invasive methods, such as colonoscopy, their results could be compromised. It is increasingly evident that no single biomarker meets all the criteria for predicting disease onset, assessing severity, gauging treatment response, and predicting recurrence and complications. The evolving landscape of research underscores the necessity for a personalized approach to inflammatory bowel diseases (IBD). This approach emphasizes tailoring diagnostic and treatment strategies to individual patient’s unique characteristics. The advent of personalized medicine in IBD holds significant promise. By leveraging insights from genetics, proteomics, metabolomics, and particularly gut microbiota research, there is potential to discover new biomarkers that better reflect the complex nature of the disease. However, while these avenues show great potential, large-scale population studies, and rigorous clinical validations are still needed to translate these findings into clinical practice. In essence, the journey towards personalized medicine in IBD underscores the imperative of integrating diverse biomarkers into a comprehensive panel, thereby paving the way for more precise and effective management strategies tailored to the individual patient’s needs.

## Figures and Tables

**Table 1:** Investigations regarding most common biomarkers in inflammatory bowel disease management.

Origin	Biomarker	Main Features	Studies
SERUM MARKERS	CRP	used in diagnosing and assessing IBD activity indicates disease severity, response to treatment, and risk of complications important role in differentiating between disease flare-ups and periods of remission persistently elevated CRP levels correlate with higher relapse risk not a disease-specific parameter not so obvious connection in UC patients	[27,29,30,31,32]
	ESR	correlation with the inflammation severity and disease activity not a disease-specific parameter influenced by age, gender, pregnancy, anemia, polycythemia, inflammatory conditions, some medications	[27,33]
	LRG	production in response to numerous cytokines, IL-6 independently more representative of intestinal inflammation than CRP correlates more accurately with clinical and endoscopic scores in active UC and CD compared to CRP correlates with hospitalizations, surgery, and clinical relapse	[21,34,35,36,37,38,39]
SEROLOGICAL ANTIBODIES	pANCA	positive test more associated with UC patients sensitivity 52%; specificity 91%—UC vs. CD	[40,41]
	ASCA	approximately 60–70% of CD patients test positive for antibodies positive in 10–15% of patients with UC; less than 5% of patients with non-IBD colitis not as valuable as a diagnostic tool; possible predictive factor in disease course	[40,42]
FECAL MARKERS	Calprotectin	used in cases of suspected IBD, IBS differentiation, disease activity monitoring, remission prediction monitoring response to anti-TNFα therapy postoperative CD recurrence prediction	[43,44,45]
	Lactoferrin	correlates well with the endoscopic and histologic disease activity utility in non-invasive disease monitoring potential cost-effective marker for assessing IBD activity particularly effective in evaluating UC activity low sensitivity scores; currently low predictive power	[46,47,48,49]
	S100A12	excellent performance in diagnosing pediatric IBD variable performance in the adult population; possible tool in differentiating IBD from functional gastrointestinal disorders	[50,51,52]

Abbreviations: CRP—C-reactive protein; CD—Crohn’s disease; UC—ulcerative colitis; ESR—erythrocyte sedimentation rate; LRG—leucine-rich Alpha-2 Glycoprotein; pANCA—perinuclear antineutrophil cytoplasmic antibody; ASCA—anti-*Saccharomyces cerevisiae* antibodies; IBS—irritable bowel syndrome.

**Table 2:** Selected investigations concerning the role of emerging molecular biomarkers in inflammatory bowel disease.

Biomarker	Study	Population	Main Results
αvβ6 antibody	Kuwanda et al. [80]	112 UC 155 HC	UC diagnosis—Se 92%; Sp 94.8% serum levels correlated with severity
	Rydell et al. [82]	59 UC 38 CD 100 IBS	UC vs. CD—Se 76.3%; Sp 79% UC vs. IBS—Se 76.3%; Sp 96%
PGE-MUM	Ishida et al. [83]	60 UC	ΔS-MES vs. ΔPGE-MUM (r = 0.518) ΔS-MES vs. ΔCRP (r = 0.444)
	Arai et al. [84]	99 UC	colonoscopic activity and remission (cut-off 21.8 μg/g·Cr; Se 81%) histological activity and remission (cut-off 17.0 μg/g·Cr; Se 82%)
miRNA	Shaker et al. [85]	35 UC 32 CD 30 HC	miRNA-675-5p: UC vs. HC—Se 85.7%; Sp 97.3% CD vs. HC—Se 88.4%; Sp 95.2%
	Luo et al. [86]	94 UC	prediction of glucocorticoid resistance: miR-16-2-3p, miR-150-5p, miR-224 5p—Sp 97.3% miR-32-5p—Se 97.4%
Oncostatin M	Cao et al. [87]	145 CD 91 UC 50 DC 32 HC	positive correlation with endoscopic and clinical disease activity combination with the fecal calprotectin: - diagnosing IBD (AUROC 0.93) - predicting therapeutic response (AUROC 0.859)
	Yang et al. [88]	818 CD 686 UC	connection with endoscopic scores, fecal calprotectin, and CRP higher levels connection with poor prognosis significantly higher OSM levels in: - non-responders vs. responders - non-remitters vs. remitters - no mucosal healing vs. with mucosal healing
BAFF	Zhang et al. [89]	37 CD 78 UC 12 IBS 44 HC	positive correlation with disease activity fecal BAFF > 325 pg/mL: active IBD vs. HC and IBS—Se 90%; Sp 96%
	Fu et al. [90]	44 CD 49 UC 27 IBS 26 HC	stronger correlation with endoscopic inflammatory scores vs. calprotectin in UC and CD IBD vs. IBS: - Fecal BAFF ≥ 227.3 pg/mL—84% Se; 100% Sp - calprotectin ≥ 50 µg/g—76% Se; 93% Sp - FOBT—65% Se; 93% Sp - BAFF + calprotectin—94% Se; 93% Sp Fecal BAFF also correlated more strongly with endoscopic inflammatory scores than calprotectin in UC and CD

Abbreviations: UC—ulcerative colitis; CD—Crohn’s disease; DC—disease controls; HC—healthy controls; IBS—irritable bowel syndrome; Se—sensitivity; Sp—specificity; PGE-MUM—prostaglandin E-major urinary metabolite; MES—Mayo endoscopic score; OSM—oncostatin M; miRNA—micro RNA; BAFF—B-cell Activating Factor.
